# The Lipoxin Receptor/FPR2 Agonist BML-111 Protects Mouse Skin against Ultraviolet B Radiation

**DOI:** 10.3390/molecules25122953

**Published:** 2020-06-26

**Authors:** Renata M. Martinez, Victor Fattori, Priscila Saito, Ingrid C. Pinto, Camilla C. A. Rodrigues, Cristina P. B. Melo, Allan J. C. Bussmann, Larissa Staurengo-Ferrari, Julia Rojo Bezerra, Josiane A. Vignoli, Marcela M. Baracat, Sandra R. Georgetti, Waldiceu A. Verri, Rubia Casagrande

**Affiliations:** 1 Departamento de Ciências Farmacêuticas, Universidade Estadual de Londrina, Avenida Robert Koch, 60, Hospital Universitário, 86038-350 Londrina, Paraná, Brazil; renatamimartinez@gmail.com (R.M.M.); prsaito@gmail.com (P.S.); carol.ingrid2@gmail.com (I.C.P.); camilla_arriero@hotmail.com (C.C.A.R.); cristinademelo@hotmail.com (C.P.B.M.); julia.bz@hotmail.com (J.R.B.); baracat1903@yahoo.com.br (M.M.B.); sangeorgetti@gmail.com (S.R.G.); 2 Departamento de Ciências Patológicas, Universidade Estadual de Londrina, Rodovia Celso Garcia Cid, Km 380, PR445, Cx. Postal 10.011, 86057-970 Londrina, Paraná, Brazil; vfattori@outlook.com (V.F.); bussmann@uel.br (A.J.C.B.); larissasferrari@gmail.com (L.S.-F.); waldiceujr@yahoo.com.br (W.A.V.J.); 3 Departamento de Bioquímica e Biotecnologia, Universidade Estadual de Londrina, Rodovia Celso Garcia Cid, Km 380, PR445, Cx. Postal 10.011, 86057-970 Londrina, Paraná, Brazil; josivignoli@yahoo.com.br

**Keywords:** SPMs, lipid mediators, resolution of inflammation, skin protection, ultraviolet damage, lipoxin, BML-111, FPR2, Nrf2, cytokine

## Abstract

Excessive exposure to UV, especially UVB, is the most important risk factor for skin cancer and premature skin aging. The identification of the specialized pro-resolving lipid mediators (SPMs) challenged the preexisting paradigm of how inflammation ends. Rather than a passive process, the resolution of inflammation relies on the active production of SPMs, such as Lipoxins (Lx), Maresins, protectins, and Resolvins. LXA4 is an SPM that exerts its action through ALX/FPR2 receptor. Stable ALX/FPR2 agonists are required because SPMs can be quickly metabolized within tissues near the site of formation. BML-111 is a commercially available synthetic ALX/FPR2 receptor agonist with analgesic, antioxidant, and anti-inflammatory properties. Based on that, we aimed to determine the effect of BML-111 in a model of UVB-induced skin inflammation in hairless mice. We demonstrated that BML-111 ameliorates the signs of UVB-induced skin inflammation by reducing neutrophil recruitment and mast cell activation. Reduction of these cells by BML-111 led to lower number of sunburn cells formation, decrease in epidermal thickness, collagen degradation, cytokine production (TNF-α, IL-1β, IL-6, TGF, and IL-10), and oxidative stress (observed by an increase in total antioxidant capacity and Nrf2 signaling pathway), indicating that BML-111 might be a promising drug to treat skin disorders.

## 1. Introduction

UV presents benefits to human health by mediating natural synthesis of vitamin D and endorphins in the skin. On the other hand, excessive exposure to UV is the most important risk factor for skin cancer and many other environmentally influenced disorders including premature skin aging [1,2]. There are three main types of UV rays: UVA (320 to 400 nm), UVB (290 to 320 nm), and UVC (200 to 290 nm). UVC does not a cause skin cancer because it does not get through our atmosphere. However, although UVB represents only 5% of UV rays, it presents more energy and is believed to be the cause of most skin cancers [1,2]. Antioxidant genes were preserved during evolution allowing the host to adapt and survive under an oxidative environment. In fact, the skin produces endogenous antioxidants including reduced glutathione (GSH) and catalase to counteract UVB-induced skin damages, which are partially related to the production of reactive oxygen species (ROS) [3,4]. Despite the effectiveness of the skin endogenous antioxidant system, excessive exposure to UV rays depletes endogenous antioxidants making the skin susceptible to the harmful actions of ROS [5,6]. Historically, humans have been exposed to UV radiation mainly through occupational exposure to sunlight. However, recreational UV exposure as a result of cosmetic purposes (tanning beds) has risen steeply over the last several years based on the misconception that a tanned look is associated with better health [7]. This activity has had a significant impact on the increasing incidence of skin cancers in the past years [7,8] and underscores the need for novel therapies to treat skin disorders alongside efforts to educate the public and government about the dangers of UV radiation.

The identification and progress on the understanding of physiopathological roles and pharmacological activity of the specialized pro-resolving lipid mediators (SPMs) challenged the preexisting paradigm on how inflammation ends. Rather than a passive process, the current concept is that SPMs, such as Lipoxins (LX), Maresins (MaR), Protectins (PD), and Resolvins (Rv) actively orchestrate the resolution of inflammation [9,10]. Consequently, the role of omega-3 and omega-6 fatty acid-derived pro-resolution mediators, such as SPMs, in the treatment and prevention of inflammatory diseases has become of interest to the academic and non-academic audience. LXA4 is an SPM derived from the metabolism of the omega-6 fatty acid, arachidonic acid [11,12,13]. Acting on ALX/FPR2, LXA4 limits inflammation by blocking neutrophil recruitment and activation, cytokine production, and oxidative stress (possibly by increasing Nrf2 signaling pathway) [11,12,13]. ALX/FPR2 is widely expressed throughout the organs such as skin and gastrointestinal tract and in immune cells, including neutrophils, monocytes, mast cells, and macrophages [14,15]. Specifically for skin inflammation induced by UVB, treatment with LXA4 [16], MaR1 [17], and RvD1 [18] ameliorate the signs of skin inflammation, indicating that this class of molecules can actively treat UVB-induced inflammation. Given that SPMs can be chemically unstable and often inactivated within tissues near the site of formation [19], stable analogs are required. BML-111 (5(S)-6(R)-7-trihydroxyheptanoic acid methyl ester) is a commercially available synthetic ALX/FPR2 receptor agonist with analgesic, antioxidant, and anti-inflammatory properties [20,21,22]. Specifically for skin inflammation, treatment with BML-111 attenuates epidermal hyperplasia and pro-inflammatory cytokine production in a model of imiquimod (IMQ)-induced psoriasis [22]. However, the effect of BML-111 in UVB-induced skin inflammation is unknown, thus verifying this possibility was the aim of the present study.

## 2. Results

### 2.1. BML-111 Reduces Neutrophil Recruitment in a Dose-Dependent Manner and an ALX/FPR2-Sensitive Manner

**Figure 1:**
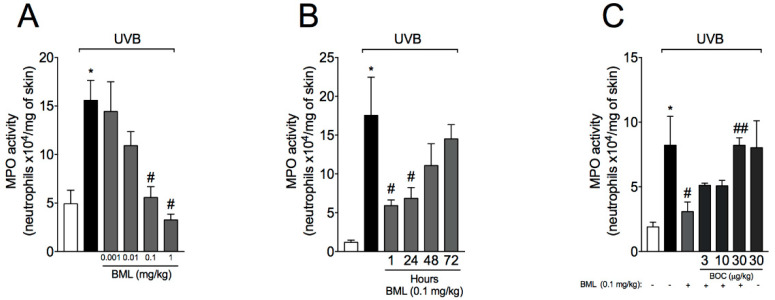
BML-111 reduces neutrophil recruitment in a dose-dependent manner and in an ALX/FPR2-sensitive manner. The effect of BML-111 in MPO activity was determined in samples dissected 12 h after the radiation (**A**–**C**). (**A**) Dose–response curve to determine the effect of BML-111. (**B**) Time–response curve to evaluate whether BML-111 presents a time-dependent effect. (**C**) Dose–response curve for BOC, an ALX/FPR2 antagonist. Results are expressed as mean ± SEM and are representative of two independent experiments. One-way ANOVA followed by Tukey’s post-test * *p* < 0.05 compared to non-irradiated group, # *p* < 0.05 compared to irradiated vehicle-treated group, ## *p* < 0.05 compared to BML-111 group.

Neutrophils are the first cells to migrate in virtually all inflammatory diseases. Therefore, we first wondered whether BML-111 reduces neutrophil recruitment induced by UVB. For that, a dose–response curve to determine the best dose of BML-111 was performed. Treatment with BML-111 at 0.1 mg/kg reduced UVB-induced neutrophil recruitment, therefore this dose was chosen for the following experiments (Figure 1A). It is now recognized that both endogenous [10,23] and isolated [16,24,25] SPMs present time-dependent effects. Specifically for skin inflammation, pre-treatment with LXA4 72 h before stimulus with UVB reduces skin edema and neutrophil recruitment [16]. Thus, it was next addressed whether BML-111 would also show this time-dependent efficacy. We chose 1 h pre-treatment regimen because we did not observe a therapeutic effect in the 72 h pre-treatment regimen (Figure 1B) as LXA4 showed [16]. These results highlight that BML-111 presents a faster effect than LXA4. We next determined whether the effect of BML-111 was related to the action on ALX/FPR2. For that, treatment with BOC (BOC-2; BOC-PHE-LEU-PHE-LEU-PHE), an ALX/FPR2 antagonist, was performed. BOC increased MPO (myeloperoxidase) activity (Figure 1C), indicating that the therapeutic effect of BML-111 is dependent on ALX/FPR2.

### 2.2. BML-111 Reduces Skin Edema and the Increase in Epidermal Thickness Induced by UVB Radiation

**Figure 2:**
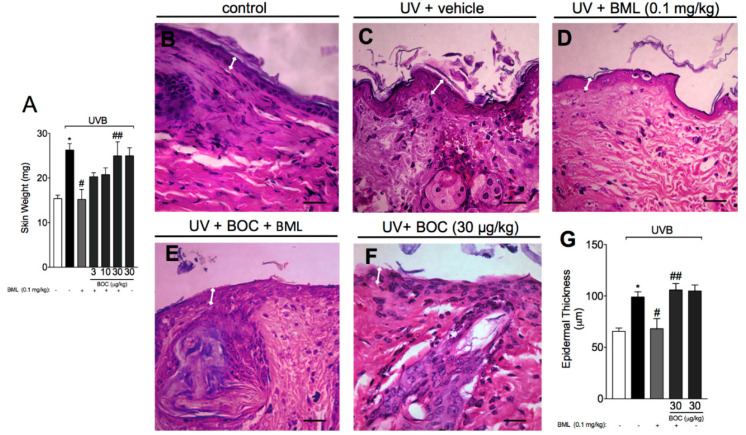
BML-111 reduces skin edema and the increase in epidermal thickness induced by UVB radiation. The skin edema (**A**) were determined in samples dissected 12 h after the radiation. The epidermal thickness was determined in samples dissected 12 h after the radiation and stained with hematoxylin and eosin (H&E). Representative images of non-irradiated control (**B**), irradiated treated with vehicle (**C**), irradiated treated with 0.1 mg/kg of BML-111 (**D**), irradiated treated with BOC and BML-111 (**E**), and irradiated treated with BOC (**F**) groups are presented. Epidermal thickness of experimental groups is presented in μm (**G**). Original magnification 40×; 100 μm. Results are expressed as mean ± SEM and are representative of two independent experiments. One-way ANOVA followed by Tukey’s post-test * *p* < 0.05 compared to non-irradiated group, # *p* < 0.05 compared to irradiated vehicle-treated group, ## *p* < 0.05 compared to BML-111 group.

Acute exposure to UVB not only induces neutrophil recruitment but also skin edema that is followed by epidermal thickening. To evaluate skin edema, samples were carefully removed and weighed, while for determination of epidermal thickness, we performed histological analysis using H&E staining. Here, we show that UVB induced an increase in skin edema (Figure 2A) and thickness of the epidermis when compared to the non-irradiated control (Figure 2B,C,G). Treatment with BML-111 reduced both skin edema (Figure 2A) and the thickness of the epidermis (Figure 2D,G). These effects were abrogated by the ALX/FPR2 antagonist BOC (Figure 2E–G).

### 2.3. BML-111 Reduces UVB-Induced Sunburn Cells

**Figure 3:**
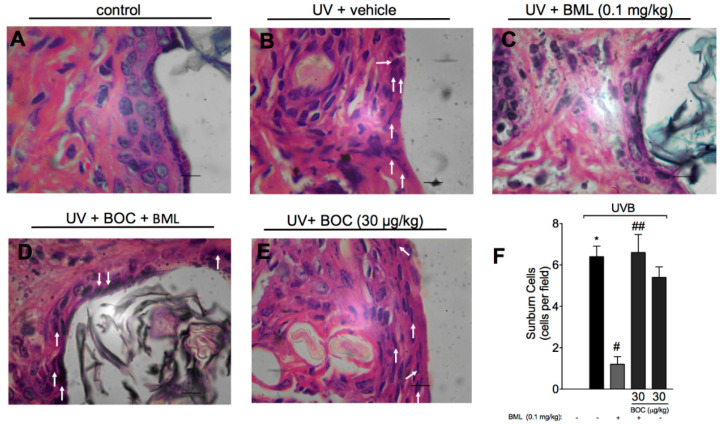
UVB-induced sunburn cells are reduced by BML-111. The number of sunburn cells was determined in samples dissected 12 h after the radiation and stained with H&E. Representative images of non-irradiated control (**A**), irradiated treated with vehicle (**B**), irradiated treated with 0.1 mg/kg of BML-111 (**C**), irradiated treated with BOC and BML-111 (**D**), and irradiated treated with BOC (**E**) groups are presented. Quantitative analysis of sunburn cells in experimental groups is presented per field in (**F**). Original magnification 100×; 100 μm. Results are expressed as mean ± SEM and are representative of two independent experiments. One-way ANOVA followed by Tukey’s post-test * *p* < 0.05 compared to non-irradiated group, # *p* < 0.05 compared to irradiated vehicle-treated group, ## *p* < 0.05 compared to BML-111 group.

Sunburn cells are keratinocytes that underwent UVB-induced apoptosis. Histologically, these cells present altered morphology as observed by chromatin condensation and eosinophilic cytoplasm. By H&E staining, we show that UVB-induced sunburn cells were reduced by treatment with BML-111 (Figure 3C,F). The therapeutic effect of BML-111 was blocked by BOC, indicating that it is sensitive to the antagonism of ALX/FPR2 (Figure 3D–F).

### 2.4. BML-111 Reduces UVB Irradiation-Induced Increase of Mast Cell Count

**Figure 4:**
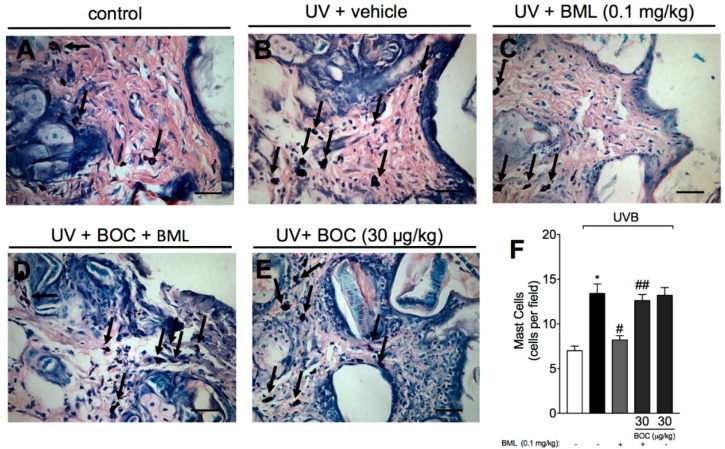
BML-111 reduces UVB irradiation-induced increase of mast cell count. Mast cells count was determined in samples dissected 12 h after the radiation and stained with toluidine blue. Representative images of non-irradiated control (**A**), irradiated treated with vehicle (**B**), irradiated treated with 0.1 mg/kg of BML-111 (**C**), irradiated treated with BOC and BML-111 (**D**), and irradiated treated with BOC (**E**) groups are presented. Mast cells count of experimental groups is presented per field in (**F**). Original magnification 40×; 100 μm. Results are expressed as mean ± SEM and are representative of two independent experiments. One-way ANOVA followed by Tukey’s post-test * *p* < 0.05 compared to non-irradiated group, # *p* < 0.05 compared to irradiated vehicle-treated group, ## *p* < 0.05 compared to BML-111 group.

After UVB irradiation, mast cells secrete mediators that trigger inflammation and recruit other leukocytes, including neutrophils [26]. Because we observed an increase in neutrophil recruitment, we next wondered whether the number of mast cell would be reduced by BML-111 as well. For that, we performed toluidine blue staining in mouse skin samples. Treatment with BML-111 reduced the number of mast cells in the skin (Figure 4C,F). This reduction was abrogated by the ALX/FPR2 antagonist BOC (Figure 4D,F), indicating that the effect of BML-111 is sensitive to BOC.

### 2.5. BML-111 Prevents UVB Irradiation-Induced Collagen Degradation

**Figure 5:**
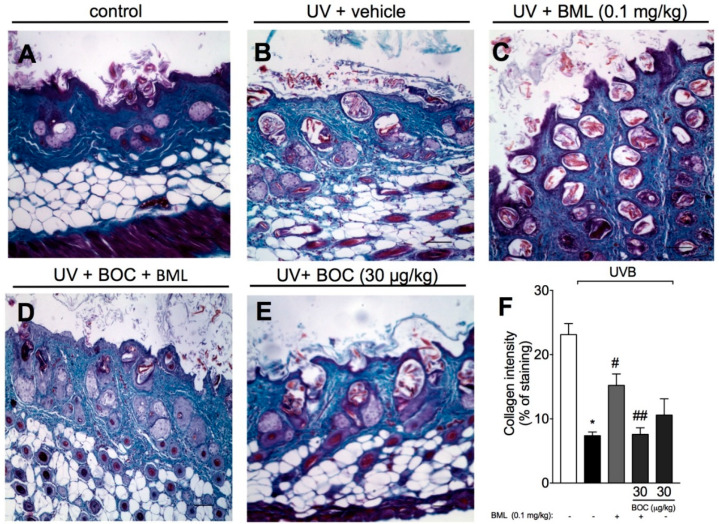
BML-111 prevents UVB irradiation-induced collagen degradation. Degradation of collagen was determined in samples dissected 12 h after the radiation and stained with Masson’s trichrome. Representative images of non-irradiated control (**A**), irradiated treated with vehicle (**B**), irradiated treated with 0.1 mg/kg of BML-111 (**C**), irradiated treated with BOC and BML-111 (**D**), and irradiated treated with BOC (**E**) groups are presented. Quantitative analysis of collagen degradation of experimental groups is presented as percentage of staining in panel (**F**). Original magnification 10×; 100 μm. Results are expressed as mean ± SEM and are representative of two independent experiments. One-way ANOVA followed by Tukey’s post-test * *p* < 0.05 compared to non-irradiated group, # *p* < 0.05 compared to irradiated vehicle-treated group, ## *p* < 0.05 compared to BML-111 group.

Photoaging is associated with the loss of collagen and other extracellular matrix proteins due to the action of enzymes called metalloproteinases, which are expressed by epidermal keratinocytes, dermal fibroblasts, and immune cells [26]. To determine collagen loss, Masson’s trichrome staining was performed because it allows the detection of collagen fibers (observed as blue color) in tissues such as skin. UVB radiation induced a significant degradation of collagen (Figure 5B,F), which was prevented by BML-111 as observed by the preservation of the blue color (Figure 5C,F). BOC reverted the effect of BML-111 (Figure 5D–F), indicating that the degradation of collagen is ALX/FPR2-sensitive.

### 2.6. BML-111 Reduces Cytokine Production During UVB-Induced Skin Inflammation

**Figure 6:**
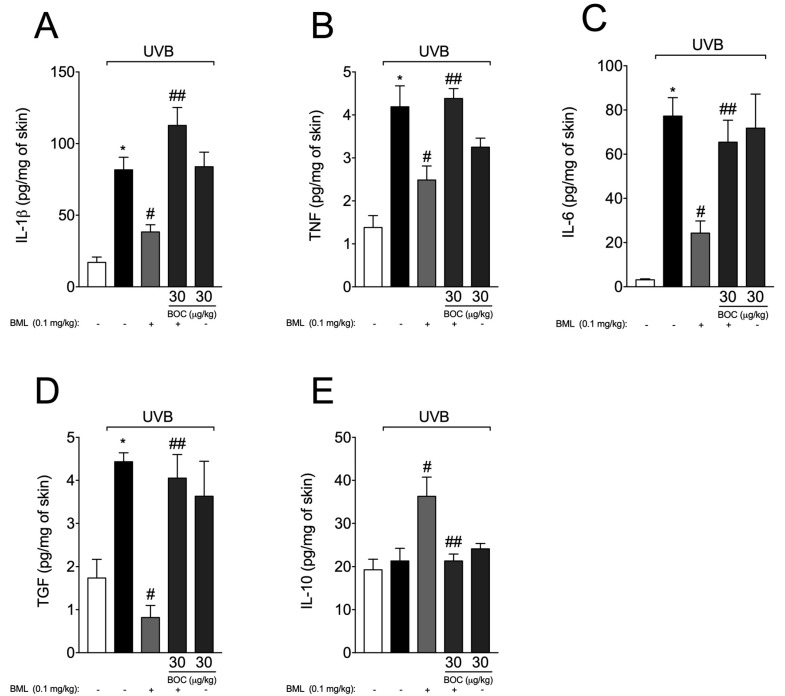
BML-111 reduces cytokine production during UVB-induced skin inflammation**.** Skin samples were dissected 4 h after the radiation to determine the levels of IL-1β (**A**), TNF-α (**B**), IL-6 (**C**), TGF (**D**), and 12 h after the radiation to determine the levels of IL-10 (**E**) by ELISA. Results are expressed as mean ± SEM and are representative of two independent experiments. One-way ANOVA followed by Tukey’s post-test * *p* < 0.05 compared to non-irradiated group, # *p* < 0.05 compared to irradiated vehicle-treated group, ## *p* < 0.05 compared to BML-111 group.

Exposure to UVB radiation leads to an increase in the production of pro-inflammatory and anti-inflammatory cytokines [16,27,28,29]. Therefore, we subsequently aimed to determine whether BML-111 could affect UVB-induced cytokine production. We observed an increase in the production of IL-1β (Figure 6A), TNF-α (Figure 6B), IL-6 (Figure 6C), and TGF-β (Figure 6D). BML-111 reduced the levels of those cytokines and increased the production of IL-10 in an ALX/FPR2-sensitive manner, i.e., treatment with BOC blocked the effect of BML-111 (Figure 6).

### 2.7. BML-111 Reduces UVB-Induced Oxidative Stress

**Figure 7:**
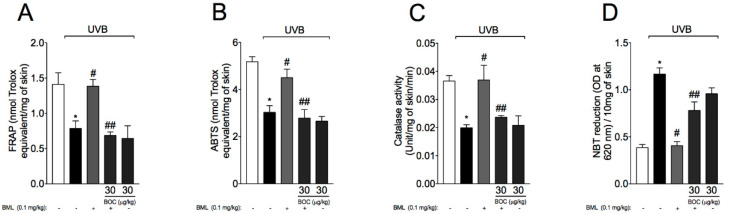
UVB-induced antioxidant capacity is restored by BML-111. Total antioxidant capacity (FRAP (**A**), ABTS (**B**)) were determined in samples dissected 12 h after the radiation. For the catalase assay (**C**) and nitroblue tetrazolium (NBT) assay (**D**) samples were dissected 2 h after radiation. Results are expressed as mean ± SEM and are representative of two independent experiments. One-way ANOVA followed by Tukey’s post-test * *p* < 0.05 compared to non-irradiated group, # *p* < 0.05 compared to irradiated vehicle-treated group, ## *p* < 0.05 compared to BML-111 group.

**Figure 8:**
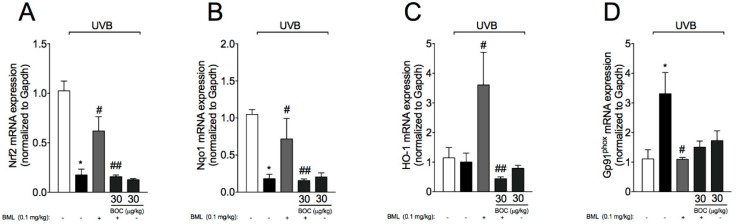
BML-111 increases mRNA expression of Nrf2 signaling pathway and reduces gp91^phox**.**^ Skin samples were dissected 4 h after the radiation to determine the mRNA expression of Nrf2 (**A**), Nqo1 (**B**), Ho-1 (**C**), and gp91^phox^ (**D**) by RT-qPCR. Results are expressed as mean ± SEM and are representative of two independent experiments. One-way ANOVA followed by Tukey’s post-test * *p* < 0.05 compared to non-irradiated group, # *p* < 0.05 compared to irradiated vehicle-treated group, ## *p* < 0.05 compared to BML-111 group.

UVB irradiation decreases antioxidant defenses as well as increasing other oxidative stress markers in the skin [16,28,30,31]. Therefore, we next evaluated the effect of BML-111 on UVB-induced oxidative stress. For that, total antioxidant capacity, enzymatic activity, and mRNA levels of antioxidant transcription factors and downstream targets were determined. We observed a reduction in total antioxidant capacity as per FRAP (Figure 7A) and ABTS assays (Figure 7B), which also reflected in lower levels of catalase (Figure 7C) and increased superoxide anion production (Figure 7D, NBT assay). Exposure to UVB radiation also reduced mRNA expression for the antioxidant transcription factor Nrf2 (Figure 8A) and downstream gene Nqo1 (Figure 8B). We also observed an increase in mRNA expression of the NADPH oxidase subunit gp91phox induced by UVB (Figure 8D). Importantly, treatment with BML-111 reduced oxidative stress by increasing antioxidant capacity (Figure 7) possibly through modulation of Nrf2 signaling pathway (Figure 8). The effect of BML-111 was prevented by BOC, indicating that it is ALX/FRP2 sensitive (Figure 7 and Figure 8).

## 3. Discussion

In this work, we demonstrate that the LXA4 receptor agonist BML-111 protects mouse skin against ultraviolet B radiation in an ALX/FPR2-sensitive manner. Treatment with BML-111 ameliorates the signs of skin inflammation induced by UVB such as increase in epidermal thickness, sunburn cells counts, and collagen degradation. Regarding immune cells, we observed a reduction in neutrophil recruitment and mast cell counts. Consequently, lower levels of cytokine production and oxidative stress were observed after treatment with BML-111. All effects were abrogated by BOC-2 (an ALX/FPR2 antagonist) further corroborating that the BML-111 effects are dependent on ALX/FPR2 agonism.

UV rays, especially UVB, are the main cause of pre-cancerous and cancerous skin lesions and are also linked to premature skin aging [1,2,32]. Occupational (sunlight) and recreational (tanning beds) exposure to UV rays are positively linked with skin cancer [33]. As tanning bed use remains prevalent, especially among young adults, and the former is inevitable, efforts toward increasing public awareness alongside with the discovery of new drugs to treat skin disorders are needed. Here, we observed that treatment with the stable synthetic ALX/FPR2 receptor agonist, BML-111, reduces the recruitment of neutrophils to the skin. After UVB radiation, mast cells are rapidly activated to release histamine and cytokines such as TNF-α [34]. These mediators are known to mediate the neurogenic phase of inflammation, which is responsible for recruiting monocytes and neutrophils [35]. Infiltrated neutrophils produce ROS, and release enzymes such as elastase that promote extracellular matrix degradation and premature skin aging [17,36,37]. Other mediators, such as the pro-inflammatory cytokine TNF-α also contribute to skin damage by inducing the formation of sunburn cells [27], which in turn can release IL-1β in a NLRP1- and NLRP3-dependent manner [38]. Herein, we demonstrated that BML-111 reduced neutrophil recruitment (as per MPO activity) and mast cell counts (Masson’s trichrome staining). Also, we showed that this ALX/FPR2 agonist reduced the levels of the cytokines IL-1β, TNF-α, IL-6, and TGF-β, while it increased IL-10 levels. Keratinocytes and macrophages are the main source of these NF-κB-dependent cytokines upon UVB radiation [39,40], which are released to control inflammatory response [41]. In a model of ovalbumin-induced asthma, treatment with BML-111 reduces NF-κB activation and the upstream adaptor molecule myeloid differentiation primary response 88 (MyD88) in the lungs and recruited immune cells [42]. Corroborating that data, in a model of psoriasis induced by IMQ, treatment with BML-111 reduced NF-κB activation and other clinical signs such as epidermal erythema, scaling, and thickening of the dorsal skin [22]. The effect of BML-111 on NF-κB activation may explain the decreased levels of cytokines observed in this work. In addition, a reduction of both mast cell counts and neutrophil recruitment by BML-111 is also an important finding that contributes to the decrease in skin inflammation and cytokine production. Considering that neutrophils and mast cells express the ALX/FPR2 receptor [14,15], it is also possible that BML-111 directly inhibited the chemoattraction of these cells. In fact, at least for neutrophils, it was shown that LXA4 and BML-111 reduce their chemoattraction [13,14,16,20,42,43].

Exposure to UV rays induces the production of ROS by keratinocytes and immune cells [31,44]. For instance, ROS activate MAPK signaling pathway leading an increase in apoptosis of skin cells [45] and activate NF-κB signaling pathway leading to an increase in pro-inflammatory cytokine production such as IL-1β, TNF-α, and IL-6 [46]. These cytokines can activate vascular endothelial cells and contribute, therefore, to the recruitment of immune cells [47,48]. siRNA targeting of the NADPH oxidase complex impairs neutrophil recruitment, indicating ROS are also implicated in the recruitment of neutrophils [49]. In addition, activation of NF-κB also regulates gp91^phox^ mRNA expression further increasing ROS production and inflammation [50]. Accordingly, treatment with PDTC (a NF-κB inhibitor) [30,51] or antioxidant molecules such as naringenin [52], dihydrocaffeic acid [45], and linalool [53] reduce UVB-induced NF-κB activation and oxidative stress, indicating a loop between ROS and this signaling pathway. Moreover, when phosphorylated, p65 NF-κB subunit competes with Nrf2 for the adaptor protein CREB binding protein (CBP) [54,55]. That mechanism dampens antioxidant response [54,55]. Therefore, the increase in UVB-induced NF-κB-dependent pro-inflammatory cytokine production observed herein may explain a reduction in antioxidant response as well (as observed by lower total antioxidant capacity and reduced mRNA expression of Nrf2 signaling pathway). Thus, drugs aiming at reducing oxidative stress are likely to be effective in reducing the signs of UVB-induced inflammation. Antioxidant molecules act mainly through two mechanisms, which are related to their scavenging ability of ROS or electron donation (direct mechanisms), or those related to activating antioxidant transcription factors, e.g., Nrf2 (indirect mechanisms) [56,57]. Here, we demonstrate that BML-111 reduced UVB-induced oxidative stress by increasing antioxidant defense, Nrf2 and the Nrf2-downstream antioxidant genes Ho-1 and Nqo1. Pro-resolving lipid mediators such as RvD1 [18] and LXA4 [16] have the ability to counteract the deleterious effects of UVB by increasing Nrf2 signaling. Our data also corroborate findings in a model of ventilator-induced lung injury, in which BML-111 increased Nrf2/HO-1 expression and reduced NF-κB activation in the lungs of rats [21]. These data and our results further indicate that an increase on Nrf2 activation (and decrease on NF-κB-dependent pro-inflammatory mediators) is a BML-111 mechanism to reduce inflammation. While we observed a reduction in ROS production in this work, SPMs are known to resolve infections by enhancing micro-organism killing without causing immunosuppression [58]. This effect over infection is related to their ability to increase bacterial killing by neutrophils and macrophages, which ultimately lead to a lower requirement of antibiotics during *E. coli* infection [58]. This effect could be particularly interesting since disruption of the skin barrier can increase susceptibility to infections [59]. In this work, we report that BML-111 restored catalase activity as well as reduced superoxide anion production and gp91^phox^ mRNA expression. We attribute the reduction of ROS and gp91^phox^ to the fact that BML-111 reduced neutrophil recruitment and mast cell activation in the skin. Thus, in this model of skin inflammation (induced by UVB, a sterile stimulus) reduction in those parameter does not indicate immunosuppressive effects, which was not either addressed or the focus of this work.

In conclusion, we demonstrated that the stable ALX/FPR2 receptor agonist BML-111 ameliorates the signs of UVB-induced skin inflammation in hairless mice by reducing neutrophil recruitment and mast cell activation. Reduction in these cells led to reduced sunburn cells, the thickness of the epidermis, collagen degradation, cytokine production, and oxidative stress. BML-111 also increased Nrf2 antioxidant signaling and IL-10 production. Therefore, BML-111 might be a promising drug to treat skin disorders.

## 4. Material and Methods

### 4.1. Animals

Experiments were performed in hairless mice (HRS/J) weighing 20–30 g, sex matched, and obtained from the Londrina State University (UEL), Paraná, Brazil. Housing was under pathogen-free conditions in cages with individual ventilation in a rack system designed for mouse with regular shaving bedding and had free access to water and food, with light/dark cycle of 12/12 h, exhausted air, and controlled temperature (22 ± 2 °C). The ethics committee/institutional review board of Londrina State University (“Comissão de Ética no Uso de Animais da Universidade Estadual de Londrina” [CEUA/UEL]) approved all procedures of this study under the process number 1447.2015.10. We confirm that all procedures/methods were performed in accordance with the relevant guidelines and regulations. Euthanasia at the end of experiments involved the sequential procedures of anesthesia with isoflurane 5% (Abbott Park, IL, USA) followed by cervical dislocation and decapitation. Mice were continuously monitored regarding welfare-related assessment before, during, and after the experiments. All efforts were made to minimize the number of animals used and their suffering.

### 4.2. Drugs and Treatment Regimen

BOC-2 (BOC-PHE-LEU-PHE-LEU-PHE, 97% purity) was purchased from Phoenix Pharmaceuticals (Burlingame, CA, USA); BML-111 (98% purity) was purchased from Sigma-Aldrich (St. Louis, MO, USA). Doses for BOC-2 and BML-111 were selected based on previous studies [60,61]. Mice received BOC-2 (intraperitoneally, in saline) 30 min before BML-111 (intraperitoneally, in saline). Where not specified, reagents were purchased from Sigma-Aldrich (St. Louis, MO, USA).

### 4.3. Irradiation Protocol

UVB lamp (Philips TL/12 RS 40W, Medical-Holand, Eindhoven, Netherlands) emission (between 270 and 400 nm, peaking at 313 nm) was on the top of the irradiation chamber and positioned 20 cm above the mice. This distance results in an irradiation of 0.384 mW/cm^2^. Irradiation was measured using a radiometer (IL 1700, Newburyport, MA, USA) equipped with UV (SED005) and UVB (SED240) sensor. The radiation dose for induction of inflammation and oxidative stress was 4.14 J/cm [3,16,62]. Mice were euthanized in specific time points after the UVB exposure according to each experiment, and the full dorsal skin was removed and stored at −80 °C to further analysis except by the samples used to determine cutaneous edema, which were weighed at the moment of collection, and to histology, which were fixed in buffered formaldehyde [26,63].

### 4.4. MPO Activity Assay

MPO colorimetric assay was used to determine neutrophil migration to the skin [32]. Samples of dorsal skin were dissected and homogenized into K_2_HPO_4_ buffer 0.05 M (pH 6.0) containing 0.5% HTAB. The homogenates were centrifuged (16,100× *g* for 2 min at 4 °C) and 30 μL of the resulting supernatant were mixed with 200 μL of 0.05 M K_2_HPO_4_ buffer (pH 6.0), containing 0.0167% *o*-dianisidine dihydrochloride and 0.05% hydrogen peroxide. Reading was performed at 450 nm (Asys Expert Plus, Biochrom). A standard curve of neutrophils was used to compare the results, which are presented as MPO activity (number of neutrophils × 10^4^ per mg of skin).

### 4.5. Skin Edema

Dorsal skin biopsy was carefully removed from euthanized mice and weighed using a precision scale [64,65]. All samples presented a constant diameter of 5 mm. Results are expressed in mg of skin tissue obtained from the weight of each sample.

### 4.6. Histopathological Analysis

Skin samples were fixed in buffered formaldehyde, embedded in paraffin, sectioned (5 μm), and stained with Masson’s trichrome stain for collagen fiber analysis (original magnification 10×). Collagen fiber intensity bundles shown in blue were analyzed by ImageJ Program (National Institutes of Health, WI, USA). Tissue sections were also stained with hematoxylin and eosin (H&E), and images were analyzed for epidermal thickness using Infinity Analyze (Lumenera1 Software, OT, Canada) (original magnification 40×). Apoptotic cells within the epidermis yielding a shrunken eosinophilic cytoplasm and a condensed nucleus were defined as sunburn cells. Sunburn cells were counted in five fields chosen at random throughout the epidermis in a conventional microscope, and the mean value of the sunburn cells obtained (original magnification 100×). Toluidine blue staining was also used to determine mast cells count in five fields chosen at random (original magnification 40×). Histopathological scores are presented together with the representative images quantifying the alterations detected between the groups. All histopathological analyses were performed by an investigator blinded to the treatment.

### 4.7. Cytokine Measurement

The levels of skin IL-1β, TNFα, IL-6, TGF, and IL-10 were measured in the supernatant using commercial enzyme-linked immunosorbent assay (ELISA) kits according to manufacturer’s instructions (eBioscience, San Diego, CA, USA). Reading was performed at 450 nm in a microplate spectrophotometer reader and the results are expressed as picograms (pg) of each cytokine/mg of skin tissue.

### 4.8. Total Antioxidant Capacity: ABTS and FRAP Assays

For both assays, skin samples were dissected and homogenized into ice-cold buffer containing 1.15% KCl. Homogenates were then centrifuged (1000× *g* in 4 °C for 10 min) [16]. A stock solution of ABTS (7mM in water) was mixed with 2.45 mM potassium persulfate (final concentration) to obtain ABTS^+^. Prior to the use, ABTS^+^ working solution was further diluted in phosphate buffer pH 7.4 to reach an absorbance of 0.8 (±0.02) at 730 nm. Briefly, the supernatant (7 μL) was added to 200 μL of the diluted ABTS^+^ solution; samples were vortex-mixed and allowed to stand for 6 min. Reading was performed at 730 nm. For the FRAP assay, the supernatants (30 μL) were mixed with the FRAP reagent. Prior to the use, FRAP reagent was prepared as follow: 0.3 mM acetate buffer pH 3.6, 10 mM TPTZ in 40 mM hydrochloride acid, and 20 mM ferric chloride. Reading was performed at 595 nm in a microplate reader. All results were compared to a standard curve of trolox (concentration ranging 0.01–20 nmol). Results are presented as nmol trolox equivalent per mg of skin tissue.

### 4.9. Catalase Assay

Catalase converts hydrogen peroxide (H_2_O_2_) in 2H_2_O + 1O_2_. To determine catalase activity, it was measured the decay on H_2_O_2_ concentration and the oxygen generation [66,67]. Skin tissue was homogenized 0.02 M EDTA (500 μL) and centrifuged twice (2700× *g*, 10 min, 4 °C). The reaction mixture contained 10 μL of sample, 160 μL of buffer Tris-HCl 1 M with EDTA 5 mM (pH 8.0), 20 μL of deionized water, and 20 μL of H_2_O_2_ 200 mM. Reading was performed at 240 nm (25 °C) and catalase activity was calculated based on the difference between the reading before and 30 s after H_2_O_2_. The catalase values were expressed as unit of catalase/mg of skin/minute.

### 4.10. Superoxide Anion Production

Superoxide anion production in the skin was measured using the nitroblue tetrazolium (NBT) reagent as described previously [16]. Fifty μL of the homogenate was incubated (37 °C, 60 min) with 100 μL of NBT (1 mg/mL) in 96-well plates. After carefully removing the supernatant, the produced formazan was solubilized by adding 120 μL of 2 M KOH and 140 μL of DMSO. Reading was performed at 620 nm. Results are expressed as NBT reduction (OD/10 mg of skin).

### 4.11. Real Time and Quantitative Polymerase Chain Reaction (RT-qPCR)

Skin samples were dissected into TRIzol reagent (Invitrogen, Carlsbad, CA, USA) and total RNA was extracted as recommended by manufacturer. The ration of the reading at 260 and 280 nm was used to determine RNA purity (between 1.8 and 2.0 for all preparations). Reverse transcription of total RNA to cDNA and qPCR were performed using GoScript™ Reverse Transcriptase and GoTaq^®^ qPCR, respectively (Promega, Madison, WI, USA) on a StepOnePlus™ Real-Time PCR System (Applied Biosystems^®^, Thermo Fisher Scientific, Waltham, MA, USA). The relative gene expression was determined using the comparative 2^−(∆∆Ct)^ method. Gapdh mRNA expression was used a reference gene to normalize data. Primer sequences: gp91phox sense 5-AGCTATGAGGTGGTGATGTTAGTGG-3, antisense 5-CACAATATTTGTACCAGACAGACTTGAG-3; and Gapdh sense 5-ATGACATCAAGAAGGTGGTG-3, antisense 5-CATACCAGGAAATGAGCTTG-3; Nqo1 sense 5′-TGGCCGAACA-CAAGAAGCTG-3′, antisense 5′-GCTACGAGCACTCTCTCAAACC-3′; Nrf2 sense: 5′-CACACGAGATGAGCTTAGGGCAA-3′, antisense: 5′-TACAGTTCTGGGCGGCGACTTTAT-3′; HO-1 sense 5′-CCCAAAACTGGCCTGTAAAA-3′, antisense: 5′-CGTGGTCAGTCAACATGGAT-3′.

### 4.12. Data Analysis

Results are presented as mean values ± standard error of the mean (SEM). Each experimental group presented 6 mice per experiment and as the experiments were conducted twice, with each group constituted of 12 mice. Statistical analysis was performed on the software GraphPad Prism 6 (GraphPad Software Inc., San Diego, CA, USA) using one-way ANOVA followed by Tukey’s *post-hoc*. When *p* < 0.05, results were taken as statistically significative.
